# When I say … productive struggle

**DOI:** 10.1111/medu.70171

**Published:** 2026-01-08

**Authors:** Sharavan Sadasiv Mucheli, Minyang Chow

**Affiliations:** ^1^ Group Clinical Education National Healthcare Group Singapore; ^2^ Lee Kong Chian School of Medicine Nanyang Technological University Singapore; ^3^ Department of Infectious Diseases Tan Tock Seng Hospital Singapore

## Abstract

If ‘productive struggle’ becomes a catch‐all for difficulty, does it retain meaning? The authors define it as supported sense‐making at the edge of competence and outline calibrated challenge, psychological safety, and guided scaffolding as the conditions that cultivate adaptive expertise. #MedEd

## 1 INTRODUCTION

In contemporary medical education, our aim is to cultivate adaptive expertise, understood as the capacity to combine efficient routine performance with flexible, innovative responses under clinical uncertainty.^1^, ^2^ ‘Productive struggle’ has emerged as one route to this goal,^3^ yet the term is often used ambiguously. In high‐workload cultures, the phrase risks becoming a rhetorical shield normalising unexamined difficulty. We therefore clarify ‘productive struggle’ as a learner‐centered construct, intended to support learning without endorsing harm.

## 2 CONCEPTUALISATION OF PRODUCTIVE STRUGGLE

When we say productive struggle, we mean the deliberate, effortful process a learner engages in when grappling with a task intentionally set just beyond their current capabilities, aided by tailored guidance. The construct has its origins in mathematics education,^4^, ^5^ and prioritises sense‐making over rapid retrieval.^4^ It is grounded in Vygotsky's Zone of Proximal Development, the optimal space for learning where a challenge is attainable with effort and support.^6^

In this view, ‘productive’ implies learner engagement that yields durable learning and prepares for future learning, rather than transient performance gains.^2^, ^7^ Because struggle is appraised within a social and affective climate, careful framing and calibrated support help learners experience effort as progress rather than threat, increasing willingness to re‐engage with difficulty.^3^, ^8^

## 3 DISTINGUISHING PRODUCTIVE STRUGGLE

Consider a student presenting a patient with a complex, multisystem illness. They are overwhelmed by disparate data and are unsure how to proceed.

In one scenario, the educator, pressed for time, provides the diagnosis and plan. The student is relieved, but the learning is superficial; they have not practised organising the information or weighing alternatives. This is *unproductive success*.^5^ In another scenario, the student is left to flounder, becoming increasingly anxious as their working memory is consumed by the stress of uncertainty.^3^ This *unproductive struggle* causes cognitive and emotional overload that hinders learning and suppresses participation.^5^ When such encounters are repeated or are experienced as threatening, it becomes a *destructive struggle*, causing learner withdrawal and disengagement.^3^, ^9^

*Productive struggle* offers a different path. The learner begins with genuine difficulty but is willing to persevere. The educator recognises the student is reaching their limits and provides a scaffold: ‘This is a complex case. Let's pause. Instead of trying to solve it all at once, what is the single most concerning problem on this list?’ Rather than correcting them, the educator stays with their thinking and uses short, targeted prompts such as, ‘What makes that stand out?’, ‘What else could explain that?’, ‘What might we miss if we only chased this?’ to help them organise the data, surface alternatives, and test their ideas.^9^ The learner remains the primary problem solver while the educator offers hints but resists taking over, preserving learner agency. The learner continues to wrestle with uncertainty and works through it within a scaffolded process that stretches rather than crushes learning.

Productive struggle sits alongside two other related constructs. *Desirable difficulties* are task‐level manipulations (e.g. spacing, interleaving and retrieval practice) that increase effort now but can yield more durable retention and discrimination.^7^ *Productive failure* is a particular sequence‐level design in which learners attempt novel problems before instruction with subsequent teaching explicitly building on their attempts.^5^ We use ‘*productive struggle*’ to name the learner's experience of sense‐making at the edge of competence under calibrated support, whether that occurs within a productive failure sequence or in much smaller cycles of supervised ward work, debriefs, or worked example study with self‐explanation. From this vantage point, desirable difficulties, productive failure and productive struggle are complementary lenses on the same family of phenomena of effortful learning.^5^, ^7^ Struggle becomes unproductive when minimal prerequisites for novices (such as some relevant prior knowledge, basic task resources and a climate where questions and errors are acceptable) are absent or when interpersonal risk is high, even if the difficulty was well intentioned.^3^, ^8^

## 4 PUTTING IT TO PRACTICE

We propose a framework of three interdependent components for productive struggle: calibrated challenge, guided scaffolding and psychological safety and equity. Calibrated challenge sets the level of difficulty; guided scaffolding shapes how learners work with that difficulty; psychological safety and equity determine who feels able to take up opportunities to struggle. Because these elements operate as a dynamic system, weakness in any one can undermine the others and can convert a learning opportunity into an ineffective or harmful experience.^3^, ^8^

### 4.1 Calibrated challenge

Design tasks aligned with the learner's zone of proximal development.^6^ The difficulty must be significant enough to require genuine cognitive effort and new thinking yet attainable to prevent excessive frustration. For learners, introduce *one difficulty at a time*. This principle is actualised through methods like problem‐based learning, complex case assignments and high‐fidelity simulations that optionally use a productive‐failure sequence in which initial struggle precedes instruction.^5^

### 4.2 Guided scaffolding

Provide timely, targeted support that guides cognition without removing the intellectual labour from the learner.^6^ A ‘warm demander’ stance, using metacognitive prompts and strategic cues, helps learners overcome impasses independently and keeps the struggle productive.^9^ (e.g. when eliciting diagnostic reasoning, ‘what is your current problem representation and what does not fit yet?’, ‘Which single finding would most change your leading diagnosis?’.)

### 4.3 Psychological safety and equity

Learners must feel secure to take interpersonal risks, such as asking questions, admitting errors and exploring uncertainty, without fear of humiliation or reprisal.^8^ Without psychological safety, challenges induce a threat response rather than fostering growth.^8^ Safety is not experienced uniformly: learners from historically marginalised or minoritized groups, by race, gender, language, disability or international status, often face greater interpersonal and identity risks^3^ when thinking aloud or challenging norms. The same ward round that feels like a stimulating challenge to one learner can feel like public exposure to another. Educators must notice whose voices are heard, whose errors are treated as learning opportunities,^9^ and, when needed, create lower‐stakes spaces (e.g. small‐group debriefs) where less secure learners can practise ‘struggling aloud’ before doing so in public.

## 5 KEEPING STRUGGLE PRODUCTIVE

Productive struggle is not hard work for its own sake. It is a pedagogical stance that values process over immediate performance. It has the power to develop the core attributes of an adaptive expert: metacognition, tolerance of uncertainty and resilience.^1^, ^2^ When done well, productive struggle reads as progress and not deficiency to learners.^3^ With careful design and precise use of the term, it cultivates deep, flexible and resilient clinical minds, the very kind modern healthcare demands.

## AUTHOR CONTRIBUTIONS

**Sharavan Sadasiv Mucheli:** Conceptualization; writing—original draft. **Minyang Chow:** Conceptualization; writing—review and editing.

## Data Availability

Data sharing not applicable to this article as no datasets were generated or analysed during the current study.
